# Dieulafoy lesion in a two-year-old boy: a case report

**DOI:** 10.1186/s13256-016-1083-4

**Published:** 2016-10-19

**Authors:** Takaki Emura, Kenji Hosoda, Shota Harai, Noboru Oyachi, Takeyuki Suzuki, Ken Takada, Koji Kobayashi, Hisatake Ikeda

**Affiliations:** 1 Department of Pediatric Surgery, Yamanashi Prefectural Central Hospital, 1-1-1 Fujimi, Kofu, 400-8506 Japan; 2 Department of Gastrointestinal Medicine, Yamanashi Prefectural Central Hospital, 1-1-1 Fujimi, Kofu, 400-8506 Japan; 3 Department of Pediatrics, Yamanashi Kosei Hospital, 860 Ochiai, Yamanashi, 405-0033 Japan

**Keywords:** Infant, Child, Dieulafoy, Gastrointestinal bleeding, Endoscope, Hemostasis

## Abstract

**Background:**

Massive gastrointestinal bleeding in children, mostly caused by esophageal varices secondary to chronic liver disease, is uncommon. Dieulafoy lesion in the gastrointestinal tract is a rare but important cause of gastrointestinal bleeding; massive bleeding from this lesion can be fatal unless adequate treatment is promptly initiated. We report a case of gastric Dieulafoy lesion in a 2-year old successfully treated with endoscopic hemoclipping.

**Case presentation:**

A 2-year-old Japanese boy was admitted to our department with sudden massive hematemesis. He had no significant past medical illness, and he was well just before the episode of hematemesis. A clinical examination revealed anemia (hemoglobin, 8.0 g/dl). The rapidly progressive anemia associated with massive hematemesis indicated the presence of an active bleeding in his upper gastrointestinal tract. We performed emergency gastroscopy under general anesthesia. The gastroscopy revealed the presence of an abnormal visible vessel with an adherent clot on the lower body of his stomach. No mucosal abnormality surrounding the lesion was noted; the lesion was thus diagnosed as Dieulafoy lesion. One hemostatic clip was placed on the Dieulafoy lesion and excellent hemostasis was obtained. He recovered without blood transfusion and was discharged 4 days post-endoscopy. He has recovered well with no recurrence of hematemesis.

**Conclusions:**

Dieulafoy lesion is rare cause of sudden massive gastrointestinal bleeding in children. Nevertheless, it should be considered a differential diagnosis, even in babies. With advances in gastrointestinal endoscopy, as both a diagnostic and therapeutic modality, laparotomy secondary to gastrointestinal bleeding from Dieulafoy lesion has decreased in pediatric cases. Our case report demonstrates the feasibility of endoscopic hemoclipping for gastric Dieulafoy lesion in a child.

## Background

Dieulafoy lesion (DL) in the gastrointestinal (GI) tract is a rare but important cause of GI bleeding; massive bleeding from this lesion can be fatal unless adequate treatment is promptly initiated [1]. Although DL has been widely described in adults, it is rarely reported among children. Here we report a pediatric case of gastric DL. To the best of our knowledge, this is the first case report in the English literature describing the successful treatment of DL in a child with endoscopic hemoclipping without blood transfusion or re-bleeding after achieving initial hemostasis.

## Case presentation

A 2-year-old Japanese boy was admitted to a regional hospital with massive hematemesis just after consuming milk. Prior to admission, he had lunch as usual and then drank milk in the evening. He was well until he experienced an episode of hematemesis. Of note, he had no significant past medical illness. On physical examination, he was pale, with a temperature of 35.9 °C, a pulse rate of 108 beats per minute, and a blood pressure of 108/52 mmHg. His height and body weight were 90 cm and 14 kg, respectively. An abdominal examination was unremarkable, and no neurological abnormalities were noted. His laboratory results showed normal hemoglobin (Hb; 12.6 g/dl), leukocyte, and platelet counts. A blood coagulation test result was also normal. However, his blood urea nitrogen (28.8 mg/dl)/creatinine (0.3 mg/dl) ratio was increased. The results of other laboratory tests (including liver enzymes) were unremarkable. He was managed conservatively with H2 blockers, intravenous fluids, and kept nil by mouth. However, on the day of admission, he developed another massive hematemesis; he was subsequently transferred to our department the following day.

**Fig. 1:**
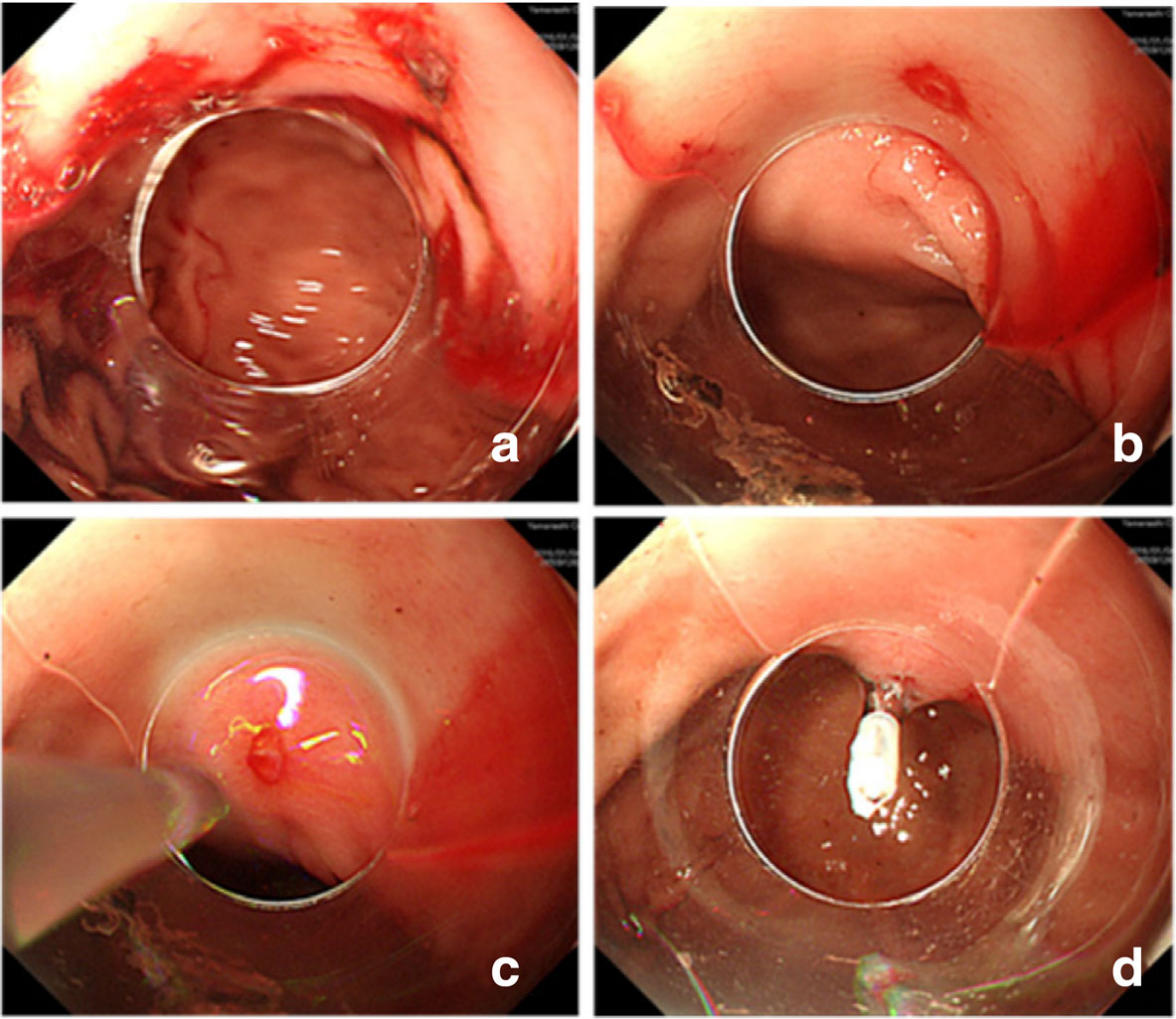
**a** Fresh blood and clotting were observed in the stomach. Gastroscopy indicated the presence of an abnormal visible vessel with an adherent clot. **b**, **c** An abnormal, oozing vessel within a minute mucosal defect and normal surrounding mucosa was visualized in the stomach. **d** The lesion was diagnosed as a Dieulafoy lesion and was managed by application of one hemostatic clip

**Fig. 2:**
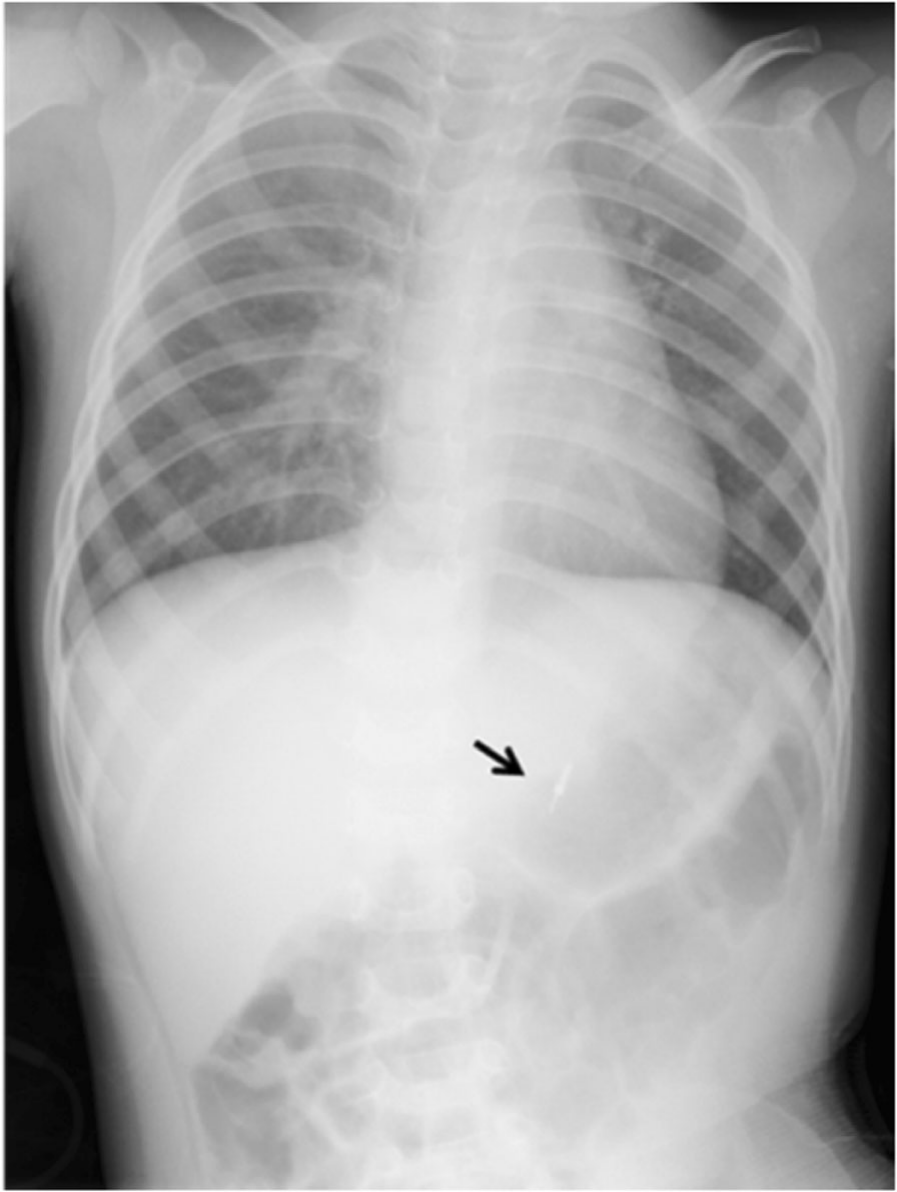
Radiogram showing the clip in the stomach (*arrow* points to the clip)

On admission in our department, his Hb had decreased to 8.0 g/dl. Blood biochemistry showed raised urea (34.1 mg/dl) with normal creatinine level (0.3 mg/dl). His rapidly progressive anemia associated with massive hematemesis indicated the presence of an active bleeding in his upper GI tract. We performed emergency gastroscopy under general anesthesia using small diameter upper endoscopes with a transparent hood over the head. During gastroscopy, fresh blood and clotting were observed within his stomach. A careful observation revealed the presence of an abnormal visible vessel with an adherent clot on the lower body of his stomach (Fig. 1). No mucosal abnormality surrounding the lesion was noted; the lesion was thus diagnosed as DL. One hemostatic clip was placed on the DL and excellent hemostasis was obtained (Fig. 1). The hemoclip (HX-610-135, Olympus) was applied with a rotatable clip-fixing device (HX-110-LR) using an endoscope (XQ240, Olympus Optical Co., Japan) with a 2.8 mm diameter accessory channel. The clip was fixed onto the lesion for a week or more (Fig. 2). Following endoscopic hemostasis, he was initiated on an intravenous proton pump inhibitor (0.7 mg/kg) daily for 2 days. He recovered without blood transfusion and was discharged 4 days post-endoscopy with a 1-month prescription of oral proton pump inhibitor. He was well at 6-months follow-up, with no recurrence of hematemesis.

## Discussion

DL of the GI tract, first described by French surgeon Dieulafoy as exulceratio simplex in 1898, is a rare but important cause of GI bleeding [2]. It is defined as a vascular abnormality consisting of a large caliber, persistent, tortuous submucosal artery. The artery fails to taper off as it reaches the mucosa, and hence has a diameter of 1 to 5 mm, which is 10 times thicker than the normal size of an artery at that level [3]. On microscopic examination, the artery protrudes through a small mucosal defect that is typically 2 to 5 mm long and does not show any other abnormality of the arterial wall. Ulceration is limited to the overlying mucosa, whereas the surrounding mucosa appears histologically normal. Up to 6 % of non-variceal bleeding in the upper GI tract is caused by DL [4]. Massive bleeding from this lesion can sometimes lead to a fatal outcome unless adequate treatment is promptly initiated [5].

Massive GI bleeding in children is uncommon and is mainly caused by esophageal varices secondary to chronic liver disease. Although DL is a well-recognized cause of GI bleeding in adults, it is rarely described in children, with only a few case reports published in the English literature [3, 6–13]. Its characteristic clinical presentation is sudden onset of painless, massive, recurrent, and intermittent hematemesis, at times associated with melena, hematochezia, and hypotension [4]. The diagnosis of DL as the source of recurrent GI hemorrhage is often delayed. DL is usually diagnosed using endoscopy. However, endoscopic diagnosis may be difficult, given the small nature of the lesion, normal appearance of the surrounding mucosa, and intermittent nature of the hemorrhage [11]. With advances in endoscopic techniques, endoscopic therapy has gradually replaced surgery and has emerged as the first option in diagnosing and managing DL [1]; initial hemostasis is achieved in approximately 95 % of cases in adults [14].

Endoscopic hemostatic procedures can be classified into three groups: (1) regional injection – local epinephrine injection and sclerotherapy; (2) thermal – electrocoagulation, heat probe coagulation, and argon plasma coagulation (APC); and (3) hemostatic clips [4, 8]. Epinephrine (dilution, 1:10,000) is repeatedly injected until cessation of bleeding is maintained [15]. Epinephrine is widely available, relatively inexpensive, simple, and comparatively safe in avoiding damage to the bowel wall. However, the use of epinephrine alone in the management of a suspected DL is not advocated given the possibility of re-bleeding [16]. Thermal endoscopic hemostasis is not preferred in children because of the risk of intestinal damage.

Several endoscopic methods have successfully treated GI DLs; in fact, it has been suggested that they were more useful and successful than injection therapy in achieving permanent hemostasis for hemorrhaging DLs [14, 15]. Theoretically, mechanical hemostasis leads to less damage to the surrounding tissue compared to other modalities [17]. If the ulcer is large and beyond the width of the clip, then achieving hemostasis with hemoclips is difficult. As such, endoscopic hemostatic clipping is feasible for bleeding from minute mucosal DLs. However, the application of a hemoclip is challenging when the angle of approach is tangential, or when the lesion is located at hard-to-reach sites, particularly in babies. In addition, prior incorrectly deployed hemoclips can hinder the accurate positioning of subsequent hemoclips. Therefore, accurate application of hemoclips, particularly the first one, is vital in DLs [18]. In our case, the clear hood over the endoscope was effective in ensuring good endoscopic field views.

## Conclusions

DL is rare cause of sudden massive GI bleeding in children. Nevertheless, it should be considered a differential diagnosis, even in babies, because massive hematemesis from gastric DL is a life-threatening symptom. DL should be suspected particularly in cases of sudden massive hematemesis with no significant past medical illness.

With advances in GI endoscopy, as both a diagnostic and therapeutic modality, the overall mortality secondary to GI bleeding from DL has decreased in pediatric cases. Our case report demonstrates the feasibility of endoscopic hemoclipping for gastric DL in a child, without any need for blood transfusion.
